# Prospective Evaluation of the Influence of Chemoradiotherapy and Stoma on Functional and Symptomatic Outcomes in Rectal Cancer Patients

**DOI:** 10.3390/cancers17122052

**Published:** 2025-06-19

**Authors:** Michael Schenker, Luiza Cristiana Bițînă, Ramona Adriana Schenker, Ana-Maria Ciurea, Alina Maria Mehedințeanu, Tradian Ciprian Berisha, Lucian Dragoș Bratu, Monica Laura Cara, Andrei Mircea Dicianu, Puiu Olivian Stovicek

**Affiliations:** 1 Department of Oncology, University of Medicine and Pharmacy of Craiova, 200349 Craiova, Romania; michael.schenker@umfcv.ro (M.S.); amciurea14@gmail.com (A.-M.C.); 2 Sf. Nectarie Oncology Center, 200347 Craiova, Romania; alina.maria591@gmail.com (A.M.M.); berishatc.sfn@gmail.com (T.C.B.); lucian.bratu@gmail.com (L.D.B.); monica.cara@umfcv.ro (M.L.C.); dicianuandrei@yahoo.com (A.M.D.); puiuolivian@yahoo.com (P.O.S.); 3 Doctoral School, University of Medicine and Pharmacy of Craiova, 200349 Craiova, Romania; 4 Department of Public Health and Management, University of Medicine and Pharmacy of Craiova, 200349 Craiova, Romania; 5 Faculty of Nursing, Târgu Jiu Subsidiary, Titu Maiorescu University, 040441 Bucharest, Romania

**Keywords:** rectal cancer, radiotherapy, chemotherapy, stoma, symptom burden, emotional functioning, quality of life

## Abstract

Background and Objectives: Rectal cancer is a major cause of morbidity and mortality worldwide, and although current therapeutic protocols have improved survival, treatment-related toxicities may significantly affect patients’ daily functioning and emotional well-being. This study aimed to prospectively assess the impact of radiotherapy with concurrent capecitabine on functional and symptomatic outcomes in patients with rectal cancer, with a particular focus on the presence of a stoma and treatment strategy. Materials and Methods: From 165 patients initially assessed, 64 were included in this study after applying eligibility criteria. All received pelvic radiotherapy (50.4 Gy in 28 fractions); 62.5% also received CAPOX chemotherapy. The quality of life was assessed using EORTC QLQ-C30 and QLQ-CR29 questionnaires administered at three time points: before treatment, mid-treatment (day 15), and post-treatment. Results: A statistically significant deterioration was observed in physical, emotional, social, and role functioning over the course of treatment, along with an increase in symptom scores for fatigue, pain, gastrointestinal, and urinary complaints. The presence of a stoma was significantly associated with worse gastrointestinal symptoms and emotional functioning. No significant differences were noted between patients with or without chemotherapy. Despite symptom worsening, global quality-of-life scores remained relatively stable. Conclusions: These findings highlight the complex interplay between treatment toxicity and patient adaptation. The presence of a stoma and other clinical or demographic factors significantly influence patients’ experience during therapy. Integrating routine assessment of functional and symptomatic burden into clinical practice could support individualized interventions aimed at maintaining daily functioning and psychological resilience during treatment.

## Simple Summary

Radiotherapy, frequently used in the treatment of rectal cancer, can significantly impact patients’ physical, emotional, and social well-being. This study examined the progression of symptoms and functional status throughout the treatment course, using validated assessment tools applied at three predefined time points during radiotherapy. The findings revealed a significant decline across all functional dimensions, including physical, emotional, and social functioning, as well as the ability to fulfill daily roles. Moreover, overall functional status worsened progressively during treatment. On the symptom scale, marked increases in fatigue, pain, urinary, and gastrointestinal complaints were observed. Comparative analysis between patients with and without a stoma indicated statistically significant differences in gastrointestinal symptoms and emotional functioning, both being more severely affected among stoma carriers. These results highlight the importance of a patient-centered therapeutic approach that integrates systematic symptom evaluation and management, emotional support, and daily functioning preservation alongside standard oncologic care.

## 1. Introduction

Colorectal cancer (CRC) ranks third in global cancer incidence, with approximately 1.9 million new cases and 935,000 deaths annually [1]. Its prevalence rises significantly after age 50 [2,3], and rectal cancer accounts for about 30% of CRC cases, with higher rates in developed countries [4]. Survival varies by stage, from over 90% in stage I to less than 15% in metastatic disease [5,6]. Although mortality has declined in high-income countries due to advances in diagnosis and treatment [7], rectal cancer remains a major global health burden. Key risk factors include advanced age [2,3] and male sex, and it is often linked to visceral adiposity, smoking, alcohol use, and dietary habits [8], as well as inflammatory bowel disease, family history, and genetic predisposition [9].

Rectal cancer significantly affects both the physical and psychological status of patients, due to its pelvic location as well as the impact of diagnosis and prognosis. Treatment involves a multimodal approach, including surgery, radiotherapy (RT), and chemotherapy (CT), each contributing not only to disease control but also to the emergence of short- and long-term side effects that may negatively influence prognosis and quality of life [10,11,12,13,14]. Radiotherapy is a central component of treatment [15,16], aiming to deliver a targeted therapeutic dose to the tumor volume while minimizing exposure to surrounding healthy tissues. Depending on the stage of the disease, it may serve a curative purpose in the neoadjuvant or adjuvant setting, or a palliative one, aimed at symptom relief and improving quality of life [17,18,19]. In the neoadjuvant context, RT is frequently combined with radiosensitizing agents such as 5-fluorouracil or capecitabine [20,21,22,23]. In metastatic disease, it may play a curative role in oligometastatic cases [24,25] or a palliative role for symptom control [26,27,28].

Surgical interventions, chemotherapy, and radiotherapy significantly influence various dimensions of quality of life. RT and CT are often associated with impairments in physical, emotional, and social functioning, with variable recovery after treatment [29]. Guren et al. [30] reported persistent gastrointestinal symptoms in 32% of patients, including chronic diarrhea (21%), anorectal dysfunction (18%), and fecal incontinence (11%), which negatively impacted global QLQ-C30 and QLQ-CR29 scores.

In locally advanced cases, the creation of a stoma, whether temporary or permanent, may become necessary to maintain digestive function and reduce postoperative complications [31]. The presence of a stoma has an additional negative impact on patients’ subjective perception of health and autonomy. D’Andrea et al. [32] reported significantly lower emotional and social functioning scores among stoma patients, particularly in older individuals and those with comorbidities. However, findings regarding the impact on gastrointestinal symptoms and pain are contradictory, highlighting the need for further research [33,34].

Despite technological advances in the planning and delivery of radiotherapy, aimed at reducing exposure to healthy tissues, adverse effects remain a major challenge in oncologic practice. Depending on the timing of onset, these effects may be acute, occurring during treatment or immediately after its completion, or chronic, manifesting months to years following radiotherapy [35]. Among acute effects, fatigue is frequently reported by patients and is perceived as a generalized exhaustion that limits the ability to carry out daily activities [36]. Gastrointestinal symptoms, such as nausea, diarrhea, and abdominal cramps, are also common, typically appearing during treatment and persisting for several weeks [37,38]. Acute cystitis, characterized by dysuria, increased urinary frequency, and hematuria, typically occurs during radiotherapy or within the first few weeks after treatment [39,40]. Chronic effects of radiotherapy are diverse and may have a significant long-term impact on quality of life [41]. At the gastrointestinal level, chronic diarrhea, malabsorption, and fistulas are frequent complications [42], while at the urinary level, hemorrhagic cystitis or urethral strictures may develop even several years after treatment completion [40].

Quality of life refers to overall well-being and includes physical, emotional, social, and functional health, as well as respect for fundamental rights [43,44,45,46,47]. To assess these dimensions in an oncological context, standardized tools have been developed for use in both research and clinical practice. Among them, the EORTC QLQ-C30 (European Organization for Research and Treatment of Cancer Quality of Life Questionnaire) is one of the most widely used instruments for oncology patients, offering a comprehensive evaluation of both functional and symptomatic status [29]. The supplementary QLQ-CR29 module, specific to colorectal cancer patients, was developed to complement the QLQ-C30 and is tailored to capture symptoms and issues commonly associated with this type of cancer [48,49,50].

Individual differences related to gender, age, and socioeconomic status may influence the perception of quality of life in patients with colorectal cancer. Baider et al. [51] reported better psychosocial compliance among men compared to women diagnosed with colon cancer. In contrast, Dibble et al. [52] found no significant differences between sexes, although women reported greater well-being in interpersonal relationships. Other studies have highlighted that younger patients experience the impact of the disease and associated stigma more intensely than older individuals [53,54], while other research did not identify a significant effect of sex or age on quality of life in colorectal or gastric cancer [55,56]. Income is an important determinant of quality of life. Ramsey et al. [57] demonstrated that patients with lower incomes reported lower scores in physical, social, and emotional well-being compared to those with higher incomes.

Although morbidity and mortality are essential parameters in evaluating therapeutic efficacy, the patient’s overall well-being and functional capacity are increasingly becoming central therapeutic goals [56]. The stage and location of the disease directly influence quality of life by determining the nature of symptoms, the treatment plan, and its duration [58,59]. In this context, Ramsey et al. [57] showed that patients in pTNM stages I and IV experienced relatively stable fluctuations in quality of life, whereas those in stages II and III initially reported a decline followed by subsequent improvement.

The creation of a stoma significantly impacts quality of life, particularly in the emotional and social domains, such as employment and sexual activity [60,61]. These effects are most pronounced in the first postoperative year, with gradual improvement over time. While physical adaptation occurs relatively quickly, psychosocial recovery varies, and support is essential. After six months, overall QoL in patients with a colostomy can become comparable to that of those without a stoma [62]. Social relationships and effective communication significantly influence quality of life, with higher satisfaction reported among patients living with family or receiving clear oncological guidance [55,56,63]. Different forms of assistance, emotional, informational, and practical, may affect QoL in distinct ways and merit further investigation. Moreover, QoL has been identified as a potential prognostic factor: lower scores are associated with shorter survival, while higher QoL predicts better outcomes regardless of disease stage [57,64,65,66].

Most studies focus on cross-sectional or retrospective assessments, without capturing the evolution of quality of life during radiotherapy with concurrent capecitabine. Prospective studies with staged evaluations remain limited in the current literature. This study expands the existing perspective by providing a sequential analysis of quality-of-life changes during radiotherapy with concurrent capecitabine. In the context of the complex impact of oncologic treatment on the functional and symptomatic dimensions of quality of life, this study aims to comparatively evaluate patients with and without a stoma, focusing on four key areas: gastrointestinal symptoms, pain, emotional functioning, and global quality of life. These dimensions are among the most frequently affected during radiotherapy with concurrent capecitabine and are clinically relevant for differentiating between patients with and without a stoma [47,48,49,50,65].

The primary objective of the study was to assess the quality of life as perceived by patients with rectal cancer during radiotherapy with concurrent capecitabine, using the standardized EORTC QLQ-C30 and QLQ-CR29 questionnaires applied at three time points during treatment. Secondary objectives included evaluating the influence of demographic variables (age, sex, and residence area), behavioral factors (smoking and alcohol consumption), clinical factors (tumor differentiation grade and disease stage), and comorbidities on the quality of life of patients undergoing radiotherapy with concurrent capecitabine, as well as identifying the factors associated with the necessity of the creation of a stoma during treatment.

## 2. Materials and Methods

### 2.1. Patient Selection and Inclusion and Exclusion Criteria

This analysis was based on a prospective, observational, and longitudinal study conducted during oncologic treatment, including 165 patients treated at the “Sf. Nectarie” Oncology Center in Craiova between June 2023 and June 2024.

Patient enrollment was conducted at the first oncology consultation, during which patients were informed about their diagnosis, disease stage, and therapeutic approach. The inclusion criteria for the study were as follows: age over 18 years; signing of the informed consent form regarding treatment and the processing of medical data for research purposes; indication for radiotherapy either with curative or palliative intent, with a total dose of 50.4 Gy, 1.8 Gy per fraction, over 28 fractions; histopathological diagnosis of rectal cancer, specifically the adenocarcinoma subtype confirmed by biopsy; radiotherapy administered concomitantly with capecitabine, with or without total neoadjuvant therapy including CAPOX. The exclusion criteria for the study included the following: indication for short-course radiotherapy; physical condition, age, or associated comorbidities that absolutely contraindicated radiotherapy, according to the decision of the multidisciplinary tumor board; treatment for another advanced cancer within the last two years; medical, social, familial, or personal conditions that could impede study participation; cognitive impairment, severely deteriorated general condition, or inability to understand or complete the questionnaires.

**Figure 1:**
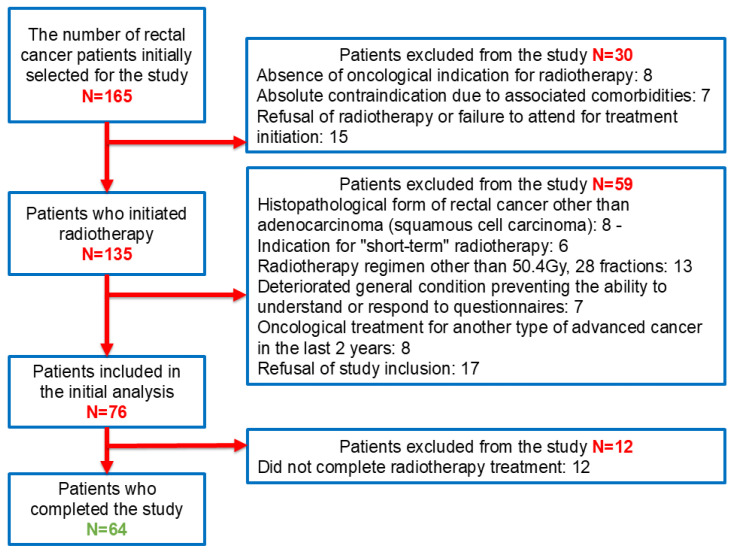
Flowchart of the patients included in the study, based on the inclusion and exclusion criteria.

Out of the total 165 patients with rectal cancer who presented for consultation, those who did not meet the indication for radiotherapy according to the international NCCN guidelines [67] and the hospital’s internal protocols, based on the decision of the multidisciplinary tumor board, were excluded (*n* = 8), as were patients with comorbidities that absolutely contraindicated treatment (*n* = 7) and those who refused radiotherapy or did not show up to begin treatment (*n* = 15). Among the remaining 135 patients who underwent radiotherapy with concurrent capecitabine, the following were excluded: patients diagnosed with squamous cell carcinoma (*n* = 8); those who received short-course radiotherapy (*n* = 6); those treated with a radiotherapy regimen different from the standard 50.4 Gy total dose, 1.8 Gy per fraction, over 28 fractions (*n* = 13); patients who were unable to understand or complete the questionnaires (*n* = 7); patients treated for another advanced malignancy in the past two years (n = 8); and those who did not consent to participate in the study (*n* = 17). A total of 76 patients were included in the study. Of these, 12 patients did not complete the 28 sessions of radiotherapy with concurrent capecitabine, resulting in a final sample of 64 patients who completed the study (Figure 1).

The study was approved by the Ethics Committee of the “Sf. Nectarie” Oncology Center in Craiova, Registration No. 481/10.05.2023. All participants provided written informed consent prior to enrollment in the study. Participation was voluntary and anonymous, without any impact on patient care.

The collected data included demographic variables (age, sex, and area of residence), level of education, behavioral factors (smoking and alcohol consumption), associated pathologies, tumor location within the rectum (lower, middle, or upper third), histopathological subtype and differentiation grade, as well as clinical and pathological TNM staging and systemic treatment administered before and during radiotherapy with concurrent capecitabine. The decision for stoma creation was made prior to the initiation of oncologic treatment, following individualized recommendations of the multidisciplinary tumor board. This decision was based on specific clinical criteria, including impaired bowel transit, tumor location (particularly low rectal tumors), advanced disease stage, and the risk of obstruction or perforation. In selected cases, a protective stoma was indicated to minimize complications during neoadjuvant therapy. Since the presence of comorbidities may further influence the quality of life in patients with rectal cancer, associated conditions were also recorded. These were classified as cardiovascular diseases, diabetes mellitus, hepatic disorders, neoplasms (other than colorectal cancer, diagnosed more than two years prior), obesity, and the co-occurrence of two chronic conditions (e.g., cardiovascular disease and diabetes mellitus). Tumor differentiation grade (G1 = well differentiated, G2 = moderately differentiated, and G3 = poorly differentiated) and tumor location within the rectum (lower, middle, or upper third) were included as clinical variables and analyzed in relation to quality-of-life outcomes.

Within the study, all patients underwent radiotherapy (total dose = 50.4 Gy, 1.8 Gy/fr, 28 fractions) concomitantly with capecitabine at a dose of 825 mg/m^2^, administered twice daily, Monday through Friday, on the days of radiotherapy. In addition, a subset of patients received chemotherapy prior to radiotherapy with concurrent capecitabine, either with neoadjuvant or palliative intent. The regimen used was CAPOX: oxaliplatin 130 mg/m^2^ IV on day 1, combined with capecitabine at a dose of 2000 mg/m^2^, administered twice daily for 14 consecutive days, with cycles repeated every 21 days. We investigated the impact of CAPOX chemotherapy on the quality of life of these patients in comparison with those who received only radiotherapy concomitant with capecitabine.

### 2.2. Questionnaire Administration Methodology

The effect of radiotherapy with concurrent capecitabine on the quality of life in patients with rectal cancer was assessed using the EORTC QLQ-C30 questionnaire [68], a cancer-specific instrument, and the EORTC QLQ-CR29 questionnaire [69], specific to patients diagnosed with colorectal cancer. Both tools were developed by the European Organisation for Research and Treatment of Cancer (EORTC) and are validated and widely used to assess quality of life in oncology patients. To capture a broad range of adverse effects that may occur during radiotherapy with concurrent capecitabine, we selected specific items from the EORTC QLQ-C30 questionnaire (21 items: 1–13, 21, 22, 25, 29–31, 42, and 43 for patients without a stoma, and 21 items: 1–13, 21, 22, 25, 29–31, 43, and 44 for patients with a stoma) and from the EORTC QLQ-CR29 questionnaire (22 items: 14–20, 23, 24, 26–28, and 32–41 for patients without a stoma, and 23 items: 14–20, 23, 24, 26–28, and 32–42 for patients with a stoma).

Thus, we obtained two questionnaires consisting of 43 items for patients without a stoma and 44 items for patients with a stoma. Each item was rated on a Likert scale from 1 to 4, where 1 = “not at all”, 2 = “a little”, 3 = “quite a bit”, and 4 = “very much”. Statistical analysis was conducted based on the raw scores obtained directly from the Likert scale in order to most accurately reflect the patients’ subjective perceptions. An exception to this scoring system was the question that differentiates the two questionnaires, namely, item number 35: “Do you have a collection bag (colostomy/ileostomy)?”, which requires a binary YES/NO response. Furthermore, six questions were focused on evaluating digestive symptoms and the psycho-emotional impact of these symptoms on the patient (in the questionnaire administered to patients without a stoma), while seven questions in the stoma-specific questionnaire aimed to assess the physical and psycho-emotional symptoms associated with the presence of a stoma. The final two items, assessing global health status and overall quality of life, deviated from the aforementioned scoring system and were rated using a visual analog scale from 1 to 7, where 1 = “very poor” and 7 = “excellent”. A higher score indicates a better general health status.

The questionnaires were administered at three predefined time points: prior to the initiation of radiotherapy with concurrent capecitabine, to assess the patient’s baseline status; on day 15 of treatment, corresponding to the period when adverse effects are frequently reported; and at the end of radiotherapy with concurrent capecitabine, after the 28th session, in order to capture the evolution throughout the entire therapeutic protocol. The questionnaires were completed by the patients on paper, under the supervision of a physician, who provided the necessary explanations and answered any questions. Completion was not subject to a time limit in order to reduce pressure on the patient and ensure the accuracy of the responses.

The functioning scale (where a lower score indicates better functioning) included the following domains: physical functioning (Q1–Q5), social role fulfillment (Q6, Q7), emotional functioning (Q29, Q32–Q34), and social functioning (Q30, Q31). The symptom scale (where a higher score indicates worsening symptoms) assessed the following: fatigue (Q8–Q10), pain (Q19, Q21, and Q38), loss of appetite and taste alteration (Q11, Q26, and Q27), nausea and vomiting (Q12 and Q13), urinary symptoms (Q14–Q17), gastrointestinal symptoms (Q18, Q20, and Q22–Q25), and hair loss (Q28). Item Q35 (“Do you have a stoma?”) was used to distinguish between patients with and without a stoma. The questionnaire for patients without a stoma assessed the following: gastrointestinal symptoms (Q36, Q37, Q39, and Q40), emotional functioning (Q41), pain (Q38), and global quality of life (Q42 and Q43). The questionnaire for colostomy patients assessed the following: gastrointestinal symptoms (Q36, Q37, Q39, and Q40), emotional functioning (Q41), pain (Q38), stoma care-related problems (Q42), and global quality of life (Q43 and Q44).

### 2.3. Data Analysis

Various statistical methods were used to analyze the data collected at the three evaluation time points in order to identify differences and assess their significance. In the preliminary stage, means and proportions were calculated for demographic and clinical variables (age, sex, area of residence, level of education, smoking, alcohol consumption, comorbidities, tumor differentiation grade, and tumor location). For each of the three evaluation time points (baseline, after 15 sessions of radiotherapy with concurrent capecitabine, and at the end of treatment), mean scores were analyzed for the functioning scales (physical, role, emotional, and social), symptom scales (fatigue, pain, loss of appetite, dysgeusia, nausea/vomiting, urinary and gastrointestinal symptoms, and alopecia), and global quality-of-life scale, separately for patients with and without a stoma. The Chi^2^ test (χ^2^) was used to assess associations between categorical variables, with statistical significance determined using the *p*-value (considered statistically significant if *p* < 0.05). The independent-samples t-test was used to compare mean scores between stoma and non-stoma groups, with the t-value indicating the statistical significance of differences between the group means, a larger absolute t-value indicating a more significant difference. ANOVA was applied to compare mean quality-of-life scores across the three treatment stages, separately for each group (with and without a stoma), indicating whether differences between time points were statistically significant. A higher F-statistic suggests significant differences between groups. Repeated-measures ANOVAs were used to assess score variation over time. Due to the limited statistical power and unbalanced subgroup sizes, adjusted analyses involving multiple predictors (e.g., mixed-effects or multivariable regression models) were not performed, as such models would have produced unstable and potentially misleading results. No prospective power analysis was performed, as this was an observational study based on consecutive eligible cases treated in our hospital during a defined period. The final sample size reflects the actual clinical population treated under the eligibility criteria.

Given the exploratory nature of the study and the relatively small sample size, corrections for multiple testing were not applied, as they would have substantially reduced the ability to detect clinically relevant differences. In addition to the tests used to assess differences over time, we calculated effect sizes (eta squared, η^2^) to quantify the magnitude of observed differences. For each mean score, 95% confidence intervals (CI 95%) were also estimated to assess the statistical stability of the results.

## 3. Results

In the analysis of the demographic and clinicopathological characteristics of the 64 patients included in the study, multiple variables were evaluated to outline the profile of the study population. The mean age of the patients was 62.6 years (median = 63, standard deviation = 10.18), with a range between 31 and 84 years. The age group distribution was as follows: 18–49 years (9.4%), 50–69 years (64.1%), and ≥70 years (26.5%) (Table 1).

Among the patients, 67.2% were male and 32.8% were female. Regarding area of residence, 56.3% came from urban areas and 43.7% from rural areas. The level of education was distributed as follows: primary (23.4%), secondary (60.9%), and higher education (15.7%). In terms of risk behaviors, 46.9% of patients were smokers, while 53.1% were non-smokers. Alcohol consumption was occasional or absent in 35.9% of patients, moderate in 53.2%, and chronic in 10.9%.

Only 37.5% of patients had no associated pathologies, while the rest presented with various comorbidities: cardiovascular diseases (34.4%), diabetes mellitus (4.7%), cardiovascular diseases combined with diabetes mellitus (10.9%), prior neoplasms (4.7%), obesity (4.7%), and hepatic disorders (3.1%). A proportion of 10.9% of patients presented with both cardiovascular conditions and diabetes mellitus. The tumor was located in the lower rectum in 31.2% of patients, in the middle rectum in 42.2%, and in the upper rectum in 26.6%. The tumor differentiation grade was G1 in 18.8% of cases, G2 in 64.1%, and G3 in 17.2%. The clinical stage distribution was as follows: IIIB (40.6%), IIIA (37.5%), IV (14.1%), IIIC (4.7%), and IIA (3.1%). Regarding chemotherapy, 79.7% of patients received the CAPOX regimen prior to radiotherapy with concurrent capecitabine, while 20.3% did not receive this treatment.

**Figure 2:**
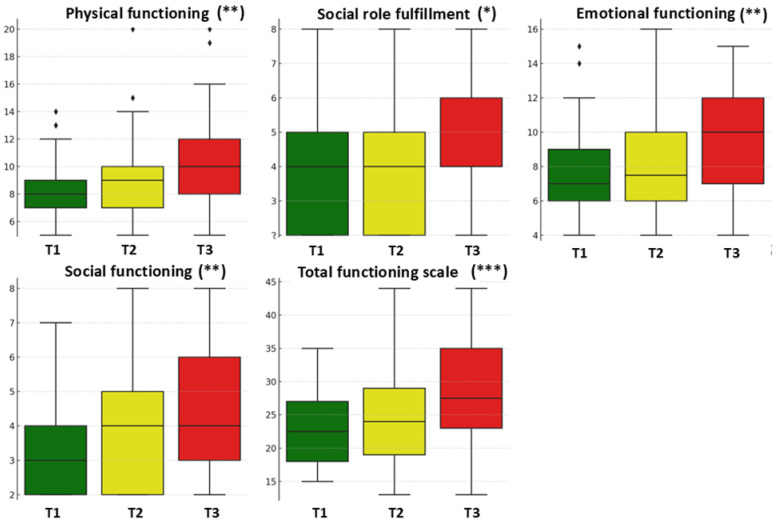
A box plot diagram comparing the evolution of functioning scale at the three evaluation moments. * *p* < 0.05, ** *p* < 0.01, *** *p* < 0.001; statistical significance based on repeated-measures ANOVA across the three time points. T1 = baseline, T2 = after 15 RT sessions, and T3 = after completing RT.

The evaluation of patients’ quality of life was based on questionnaires administered at three stages of treatment (baseline, after 15 RT sessions, and after the final RT session) and included both functioning and symptom scales. Physical functioning significantly deteriorated over the course of treatment, with mean scores increasing from 8.31 (baseline) to 8.88 (after 15 RT sessions) and 10.16 (final session), as higher scores reflect greater functional impairment (*p* = 0.000540, ANOVA = 7.83) (Table 2). Scores for role functioning increased significantly from 3.86 to 4.02 and 4.70 (*p* = 0.021256, ANOVA = 3.93), suggesting a gradual decline in patients’ ability to perform daily and social activities. Emotional functioning also significantly worsened during treatment, with mean scores rising from 7.78 to 8.17 and 9.55 (*p* = 0.001304, ANOVA = 6.88), indicating progressively greater emotional impairment. Social functioning scores increased from 3.30 at baseline to 3.67 after 15 RT sessions and 4.30 at the end of treatment (*p* = 0.001588, ANOVA = 6.67), reflecting growing difficulties in patients’ ability to interact and integrate socially. The significant increase in total functioning scale scores from 23.25 to 24.73 and 28.70 (*p* = 0.000055, ANOVA = 10.34) indicates an overall deterioration of physical, emotional, and social status throughout the treatment period (Figure 2).

The analysis of effect size over time (η^2^) and 95% confidence intervals (CI 95%) for functional scores confirmed the statistical significance indicated by the *p*-values. η^2^ values ranging from 0.0587 to 0.1410 suggest moderate to large effect sizes, indicating that changes in functional scores over time are not only statistically significant but also relevant in terms of magnitude. Such changes are particularly important in quality-of-life research, where even small to moderate effect sizes can have meaningful clinical implications. The 95% confidence intervals for the mean scores did not overlap between baseline and final assessment, supporting the stability of the observed differences and reinforcing both the statistical validity and clinical relevance of functional decline during treatment.

**Figure 3:**
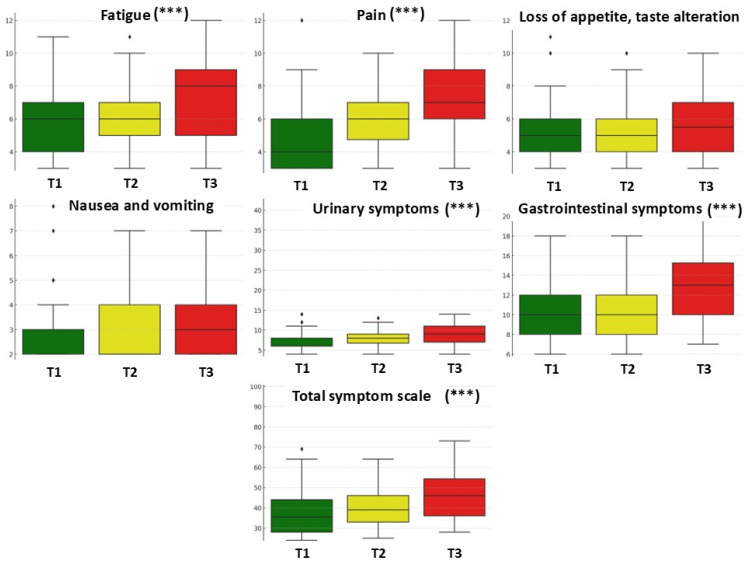
A box plot diagram comparing the evolution of symptom scale at the three evaluation moments. *** *p* < 0.001; statistical significance based on repeated-measures ANOVA across the three time points. T1 = baseline, T2 = after 15 RT sessions, and T3 = after completing RT.

The analysis of symptom scores throughout treatment included fatigue, pain, loss of appetite and taste alteration, nausea and vomiting, urinary and gastrointestinal symptoms, hair loss, and the total symptom scale. Fatigue significantly increased, with mean scores rising from 5.91 (baseline) to 6.19 (after 15 RT sessions) and 7.48 (final session) (*p* < 0.001, ANOVA = 8.75) (Table 3). Similarly, pain increased from 4.69 to 5.98 and 7.30 (*p* < 0.001, ANOVA = 25.67) (Figure 3). Loss of appetite and taste alteration remained relatively constant (5.22, 5.13, and 5.83, respectively), showing no significant differences (*p* = 0.076, ANOVA = 2.61). Nausea and vomiting varied insignificantly between 2.98, 2.92, and 3.34 (*p* = 0.163, ANOVA = 1.83), suggesting minimal impact of treatment on this symptom. Urinary symptoms progressively worsened from 6.89 to 7.89 and then 9.42 (*p* < 0.001, ANOVA = 9.48), while gastrointestinal symptoms increased from 10.20 to 10.77 and 12.98 (*p* < 0.001, ANOVA = 13.92), indicating a significant deterioration during treatment. Hair loss remained stable with scores of 1.17, 1.22, and 1.25 (*p* = 0.7537, ANOVA = 0.28), with no significant differences between time points. The total symptom scale, which summarizes all clinical manifestations, increased significantly from 37.06 to 40.09 and 47.61 (*p* < 0.001, ANOVA = 15.42), reflecting a generalized worsening of symptoms over the course of treatment.

The results indicate that treatment significantly exacerbates fatigue, pain, urinary and gastrointestinal symptoms, as well as the total symptom scale. In contrast, loss of appetite, taste alteration, nausea, vomiting, and hair loss did not show significant variation across treatment stages. These findings contribute to a better understanding of the therapeutic impact and support the optimization of symptom management.

The distribution of patients analyzed shows that 79.7% (51 patients) did not require a stoma, while 20.3% (13 patients) had an indication for this intervention, allowing for a comparative analysis of these two subgroups in terms of quality-of-life scores. Correlation analysis, using χ^2^ significance thresholds corresponding to degrees of freedom (df) and *p* < 0.05, revealed a significant association between the need for a stoma and several clinical and demographic factors.

Age was the most relevant predictor (χ^2^ = 64.42, df = 2, threshold χ^2^ = 5.99, *p* < 0.001), indicating an increased risk of requiring a stoma with advancing age. Sex was also significant (χ^2^ = 8.66, df = 1, threshold χ^2^ = 3.84, *p* = 0.003), with a higher predisposition among male patients.

Level of education (χ^2^ = 9.70, df = 2, threshold χ^2^ = 5.99, *p* = 0.008) showed a greater risk among patients with only primary education, possibly due to limited healthcare access or health-related behaviors.

The analysis of effect sizes (η^2^) and 95% confidence intervals (CI 95%) confirms the clinical relevance of symptoms that showed statistically significant variation, particularly pain (η^2^ = 0.2136), gastrointestinal symptoms (η^2^ = 0.1284), and fatigue (η^2^ = 0.0848). The absence of overlap between confidence intervals at baseline and final assessment supports the consistency of these changes. For the other symptoms, low η^2^ values and overlapping confidence intervals indicate minimal variation without clinical relevance.

Disease stage (χ^2^ = 14.19, df = 4, threshold χ^2^ = 9.49, *p* = 0.0067) confirmed that patients diagnosed at advanced stages (IIIC and IV) had a significantly higher probability of requiring a stoma. In addition, three variables showed statistically significant associations with stoma presence: age (χ^2^ = 64.42, df = 2, threshold χ^2^ = 5.99, *p* = 0.0004), sex (χ^2^ = 8.66, df = 1, threshold χ^2^ = 3.84, *p* = 0.0032), and level of education (χ^2^ = 9.70, df = 2, threshold χ^2^ = 5.99, *p* = 0.0078). These findings suggest that older patients, males, and individuals with lower educational attainment are more likely to require a stoma, underscoring the role of sociodemographic factors in treatment planning. In contrast, no significant associations were identified for area of residence (*p* = 0.5721), smoking (*p* = 1.0), alcohol consumption (*p* = 0.7547), tumor location (*p* = 0.5490), chemotherapy (*p* = 0.5261), associated pathologies (*p* = 0.3768), or tumor differentiation grade (*p* = 0.1270), suggesting a limited influence of these factors on the need for a stoma (Table 4). These findings are important for understanding the factors involved in the need for stoma creation and have implications for the individualization of therapeutic strategies. In a larger sample, associated pathologies and tumor differentiation grade may become statistically significant.

This study also analyzed the mean scores of patients with and without a stoma at the three stages of treatment (baseline, after 15 RT sessions, and after the final session). For patients without a stoma, the evaluation included gastrointestinal symptoms, emotional functioning, pain, and global quality of life, while for those with a stoma, stoma care-related issues were also assessed. Among patients without a stoma, gastrointestinal symptoms worsened, with scores increasing from 7.14 at baseline to 9.35 after the final session (*p* = 0.000151, ANOVA = 9.34), indicating a statistically significant aggravation (Table 5). Emotional functioning increased from 1.94 to 2.39, which was not statistically significant, but approached the threshold (*p* = 0.05345, ANOVA = 2.99). Pain showed a significant increase over the course of treatment, rising from 1.43 to 2.75 (*p* < 0.001, ANOVA = 36.01). On the global quality-of-life scale, scores decreased from 10.24 to 9.16, but this change was not statistically significant (*p* = 0.1125, ANOVA = 2.22), suggesting that treatment did not substantially influence this parameter.

For patients without a stoma, the analysis of effect sizes (η^2^) and 95% confidence intervals (CI 95%) confirmed the statistical results. A large effect was observed for pain (η^2^ = 0.2483) and a moderate effect for emotional functioning (η^2^ = 0.0663), indicating that the worsening of these symptoms is not only statistically significant but also clinically relevant. The confidence intervals for mean scores at baseline and final assessment did not overlap for these dimensions, supporting the consistency and stability of the observed differences. In the subgroup of patients with a stoma, the η^2^ analysis revealed a large effect for gastrointestinal symptoms (η^2^ = 0.3593) and a moderate effect for emotional functioning (η^2^ = 0.1795), supporting the clinical relevance of these changes. The confidence intervals for mean scores did not overlap between the initial and final time points for these scales, suggesting that the progression of symptoms was real and consistent throughout the course of treatment.

For patients with a stoma, the analysis of mean scores throughout treatment revealed a significant worsening of gastrointestinal symptoms, with an increase from 6.23 at baseline to 10.38 after the final session (*p* = 0.000331, ANOVA = 10.09) (Table 5). Emotional functioning moderately deteriorated, with scores rising from 2.38 to 3.15; this variation was statistically significant (*p* = 0.0284, ANOVA = 3.94). In contrast, pain did not show significant differences, with scores increasing slightly from 1.62 to 2.31 (*p* = 0.1057, ANOVA = 2.39). Stoma care-related problems remained relatively stable, with scores ranging from 2.00 to 2.38, without a significant difference (*p* = 0.5496, ANOVA = 0.61). Similarly, the global quality-of-life scale showed a slight decrease from 10.31 to 9.46, but this change was not statistically significant (*p* = 0.6924, ANOVA = 0.37).

When comparing patients with and without a stoma, the results show that, for those without a stoma, gastrointestinal symptoms and pain increased significantly, while emotional functioning and quality of life were not significantly affected. In patients with a stoma, only gastrointestinal symptoms and emotional functioning varied significantly, whereas pain, stoma care, and quality of life were not influenced by treatment. These findings highlight the differences in how treatment affects patients’ quality of life and emphasize the need for personalized symptom management strategies based on the presence or absence of a stoma.

We conducted a comparative analysis of mean scores for patients with and without a stoma, evaluating gastrointestinal symptoms, emotional functioning, pain, and the global quality-of-life scale. The results of the independent-samples t-test showed that for gastrointestinal symptoms, the mean scores were 8.15 for patients without a stoma and 8.18 for those with a stoma (t = −0.05689, *p* = 0.954832), indicating no significant difference between the two groups (*p* > 0.05).

Regarding emotional functioning, patients with a stoma had a significantly higher mean score (2.59) compared to those without a stoma (2.15), suggesting a more pronounced negative emotional impact (t = −2.5444, *p* = 0.01362). For pain, the mean scores were similar between the two groups (2.13 for patients without a stoma and 2.05 for those with a stoma), with no statistically significant difference (t = 0.47963, *p* = 0.63322, *p* > 0.05). The global quality-of-life scale had mean values of 9.60 for patients without a stoma and 10.03 for those with a stoma, with no significant difference (t = −0.83921, *p* = 0.404852, *p* > 0.05). These results indicate that patients with a stoma experience significantly greater emotional impairment, while gastrointestinal symptoms, pain, and quality of life do not differ between the two groups.

No statistically significant differences were identified between patients with and without chemotherapy for any of the functioning scores across the three treatment phases, as all *p*-values were greater than 0.05 (Table 6). Similarly, none of the symptom scales revealed statistically significant differences between patients who did or did not receive chemotherapy. However, these results should be interpreted with caution, as the lack of statistical significance does not imply equivalence between groups or absence of clinical effects (Table 7).

## 4. Discussion

In this study, we included the analysis of patients’ demographic data to explore the impact of these variables on the perception and assessment of quality of life in the context of radiotherapy with concurrent capecitabine among patients diagnosed with rectal cancer. The analyzed patient cohort was predominantly male, with most patients aged between 50 and 69 years. These findings are consistent with international epidemiological trends indicating a higher incidence of colorectal cancer among middle-aged and older men [70].

Depending on age, patients may have different perceptions and responses to treatment, which in turn can influence quality of life. Older patients may exhibit lower tolerance to treatment-related side effects and often present with comorbidities that further affect their quality of life. Over 90% of older adults with cancer have one or more comorbidities, complicating the management of oncologic treatment and affecting its outcomes [71]. Additionally, men and women may perceive and report symptoms and the impact of treatment on their general well-being differently. Women have reported more physical symptoms, including nausea and vomiting, insomnia, and loss of appetite, compared to men [72]. The majority of patients in this study were from urban areas, which may reflect easier access to specialized medical services and social support, and consequently a higher likelihood of earlier diagnosis and treatment. Patients from rural areas may have more limited access to support and care services, which can negatively affect quality of life [73].

Regarding behavioral risk factors, nearly half of the patients in the study were smokers, and approximately two-thirds reported alcohol consumption. These behaviors are major risk factors for multiple complications and may exacerbate treatment-related symptoms. Alcohol consumption and smoking are well-recognized as aggravating factors during radiotherapy with concurrent capecitabine, negatively influencing treatment response and the quality of life of oncology patients [74]. Assessing these factors allows us to determine whether there is a relationship between unhealthy habits and the perception of quality of life.

The majority of patients had a medium level of education, which can significantly influence their ability to understand the diagnosis, therapeutic options, and management of treatment-related side effects, as well as their participation in medical decision-making and adherence to treatment [75]. Patients with a higher level of education may have a better understanding of the diagnosis, treatment options, and adverse effects, which can contribute to maintaining a balanced perspective on quality of life. Higher education is also frequently associated with enhanced skills in seeking and using medical information, as well as easier access to psychological and social support resources. Consequently, these patients may develop more effective coping strategies when facing the challenges of oncologic treatment. Educational level, often correlated with socioeconomic status, can influence patients’ ability to access information, psychological support, and the material resources necessary for effective psychosocial adaptation to treatment [76].

Comorbidities were present in more than half of the patients, particularly cardiovascular diseases and diabetes mellitus, factors that may negatively influence disease progression and the patients’ quality of life. The presence of comorbidities has been associated with lower levels of physical functioning and a negative perception of general health status among oncology patients [77,78]. These clinical and demographic factors provide a relevant framework for interpreting the recorded changes in the functional and symptomatic dimensions of quality of life.

The results related to physical functioning indicated a progressive decline, reflected by the increase in mean scores from baseline to the end of treatment. This trend suggests a continuous deterioration in patients’ ability to carry out routine activities such as walking, climbing stairs, and performing other moderate physical tasks. The observed pattern reflects the impact of acute adverse reactions associated with radiotherapy administered concomitantly with capecitabine on overall physical functioning.

During oncologic treatment, patients may experience a gradual decrease in energy levels and a decline in overall functional capacity, which limits their ability to perform daily physical activities [79]. This impairment is particularly pronounced among older patients, who exhibit increased vulnerability to radiotherapy with concurrent capecitabine, resulting in a more severe decline in physical functioning and in the ability to fulfill social or occupational roles [80].

Regarding role functioning, mean scores increased significantly across all three assessment time points, indicating a progressive deterioration in patients’ ability to adapt socially and to assume daily responsibilities. This trend reflects the negative impact of oncologic treatment, which may limit patients’ active engagement in professional, familial, and social life, affecting their capacity for social integration and adaptation, and amplifying the disease’s impact on daily activities.

At the end of radiotherapy with concurrent capecitabine, significant deteriorations in general health status were observed, accompanied by worsening symptoms such as nausea, vomiting, and loss of appetite, indicating the cumulative negative impact of treatment on the patient’s overall condition [81]. Throughout the course of treatment, patients’ emotional functioning was significantly affected. This negative progression reflects a loss of emotional balance and a reduction in social engagement, impairing the ability to adapt to professional and family life. This aspect is particularly relevant, as patients may experience psychological changes even before the initiation of radiotherapy with concurrent capecitabine, manifested by worry, fear, sadness, depression, nervousness, and loss of interest in daily activities [82].

Regarding social functioning, mean scores gradually increased over time, indicating a decline in patients’ ability to maintain interpersonal relationships and participate in social activities. This reduced social engagement may be driven by accumulated fatigue, persistent physical symptoms, and heightened emotional distress during treatment. Social functioning in patients treated with radiotherapy with concurrent capecitabine for rectal cancer may remain impaired in the long term due to the persistence of anorectal, urinary, or sexual dysfunctions [47,83,84].

The total functioning scale, which integrates physical, role, emotional, and social functioning dimensions, showed a progressive increase in mean scores from the first to the final session of radiotherapy with concurrent capecitabine, indicating a global impairment in patients’ functional capacity. The observed differences are statistically significant and point to a consistent decline in overall functioning as treatment progresses.

During radiotherapy with concurrent capecitabine, a reduction may occur across all functioning domains, affecting multiple aspects of quality of life [85]. However, some of these changes may prove to be temporary, as patients are often able to tolerate treatment despite significant functional and symptomatic impairments [86].

Pain scores showed a significant increase, indicating a progressive worsening of this symptom. This trend is consistent with findings from the literature, which emphasize the cumulative harmful effects of radiotherapy with concurrent capecitabine on pelvic structures, contributing to an intensification of pain experienced during therapy [87,88,89].

With regard to symptom score analysis, the successive increase in mean scores for fatigue indicates a significant rise in fatigue levels throughout treatment. Fatigue intensification among patients with rectal cancer is common, particularly during the first month of treatment, with variations in severity over the course of therapy [90], or a progressive, linear trajectory, as reported in another study [91]. This progression is supported by evidence indicating a gradual increase in fatigue during radiotherapy with concurrent capecitabine [85], as well as its association with deteriorated sleep quality and circadian rhythm disruption [92]. In some cases, fatigue onset occurred even before the initiation of radiotherapy with concurrent capecitabine [90], where causes were independent of oncologic treatment. Predictive factors for fatigue have included uncontrolled pain or diarrhea [91], as well as the structure of the intestinal microbiota [93,94].

Mean pain scores also increased significantly by the end of treatment, indicating that pain becomes more intense over the course of therapy. In addition to the pain associated with the emotional burden of the diagnosis [95] and the manifestations of the disease itself, radiotherapy with concurrent capecitabine exacerbates this symptom through inflammatory reactions in the irradiated area, affecting the rectal mucosa and pelvic structures. These effects are compounded by bowel and urinary dysfunction, which further intensify pain [88]. Throughout treatment, pain demonstrated a trend of increasing intensity and was associated with higher levels of fatigue [96]. This worsening was more frequently reported among younger patients, suggesting a potentially greater vulnerability in this population group [86].

Mean scores for loss of appetite, nausea and vomiting, and taste alteration varied but did not show statistically significant differences across treatment stages. Appetite loss as an adverse effect of radiotherapy with concurrent capecitabine has been described as being associated with the treatment’s impact on the digestive mucosa and overall condition [97]. An association has also been identified between elevated fatigue levels and the exacerbation of gastrointestinal symptoms, particularly nausea and vomiting, among oncology patients [96]. However, in some patients, no increase in nausea or pain was observed after the completion of radiotherapy with concurrent capecitabine [97].

Radiation-induced tissue damage has consequences for the urinary system through fibrosis and neovascularization. Neovascularization plays a major role in the onset of radiation cystitis and hemorrhagic cystitis, while fibrosis of the corpus spongiosum and occlusion of the urethral lumen are major causes of urethral strictures following radiotherapy with concurrent capecitabine [98]. The progressive increase in mean scores for urinary symptoms suggests a worsening of these symptoms during radiotherapy with concurrent capecitabine. These adverse reactions may occur during treatment [84,99] or may persist long-term after its completion in the form of residual urinary symptoms such as dysuria, increased urinary frequency, or incontinence [83,100].

Alongside urinary symptoms, gastrointestinal symptoms also increased significantly during treatment. Patients receiving radiotherapy with concurrent capecitabine to the lower gastrointestinal tract may experience abdominal cramps and pain, as radiation can induce inflammation or alter the composition of the intestinal microbiota [93,94]. Diarrhea is the most common short-term adverse effect of radiotherapy with concurrent capecitabine in this region, caused by inadequate water absorption, while some patients may experience pain during defecation due to skin inflammation [83,84,100]. The total symptom scale showed a highly statistically significant increase in mean scores from baseline to the final session, indicating a notable intensification of overall symptoms during treatment. Thus, it is important to emphasize the need for a comprehensive approach to symptom management in order to improve patients’ quality of life, particularly when symptom exacerbation occurs in younger patients [86].

According to the results of this study, 20.3% of patients had a stoma, while 79.7% did not. Patient age was significantly correlated with the presence of a stoma, suggesting that older individuals are more likely to require stoma creation. A significant correlation between sex and stoma presence also indicated that men are more likely than women to have a stoma. Additionally, patients with only primary education had a higher likelihood of requiring a stoma compared to those with secondary or higher education. This may reflect differences in access to medical information and resources, or disparities in socioeconomic status. Increased anxiety and negative perceptions of quality of life among some stoma patients may be explained by the psychological impact of surgery and the challenges of postoperative adaptation. This emotional vulnerability may amplify the overall impact of oncologic treatment on psychosocial functioning [101,102].

Colostomy creation was significantly more frequent among rectal cancer patients without higher education, a statistically significant difference, and five-year survival was higher among patients with a higher educational level, suggesting a potential influence of educational status on therapeutic decisions and prognosis [103]. The results obtained highlight the negative impact of stoma presence on emotional and social functioning, emphasizing the importance of counseling and education at this stage. A clear benefit of education lies in strengthening coping and self-management skills, thus contributing to more effective patient adaptation. The variability in quality-of-life responses also points to the need for additional assessments using standardized methods in order to guide future clinical interventions [104].

Disease stage was significantly correlated with stoma presence, with patients in stages IIIC and IV being more likely to require a stoma. However, no significant correlations were found between stoma presence and area of residence, smoking, alcohol consumption, associated pathologies, tumor location, degree of differentiation, administration of chemotherapy, or type of chemotherapy administered. This suggests that these factors do not significantly influence the likelihood of requiring a stoma. Among patients without a stoma, the results showed a significant increase in gastrointestinal symptom scores, indicating a worsening of symptoms during treatment, as well as a significant increase in pain scores, reflecting intensifying pain throughout therapy. With regard to emotional functioning and the global quality-of-life scale, the score changes were not statistically significant.

For patients with a stoma, a significant increase in gastrointestinal symptom scores was observed, indicating a worsening of symptoms during treatment. However, regarding pain, unlike in patients without a stoma, only a non-significant increase in scores was recorded, indicating insufficient variability to be considered statistically significant. Non-significant increases were also reported regarding stoma care, suggesting that stoma management was not significantly affected by the treatment. In terms of emotional functioning, the significant increase in scores reflects a marked decline in emotional well-being. The global quality-of-life scale showed a non-significant decrease in scores, indicating that treatment did not have a significant impact on perceived quality of life. This finding should be interpreted with caution in light of the observed deterioration in physical, emotional, and social functioning, as well as the intensification of specific symptoms. The absence of a significant change in global QoL does not necessarily indicate the preservation of well-being, as clinically relevant impairments may still be present. These findings are particularly relevant for clinical counseling and may inform shared decision-making, especially in the context of invasive therapeutic options such as stoma creation. Incorporating such data into clinical discussions may help adjust patient expectations and support individualized care planning. However, this apparent stability in global QoL scores may reflect psychological adaptation mechanisms activated during cancer treatment. These may include adjustment of expectations, selective focus on perceived benefits of therapy, reframing of the illness context, or a sense of active involvement in the therapeutic process. As a result, patients may maintain a globally favorable perception of quality of life, even when experiencing quantifiable deterioration in functioning or symptoms. Distinguishing between these levels of perception is important for understanding the observed discrepancy between global and domain-specific quality-of-life scores.

The impact of a stoma on quality of life may lessen over time, as many patients develop effective coping mechanisms within 2–3 months after stoma creation, particularly when receiving appropriate psychological and educational support [62]. A self-efficacy-based intervention conducted over a three-month period significantly improved quality of life among patients with permanent colostomies [105]. These findings underscore the importance of ongoing support and education in the postoperative period to facilitate adaptation and improve long-term outcomes. Based on these findings, there is a clear need for a proactive approach that includes pre-treatment education about life with a stoma, personalized nutritional counseling for gastrointestinal symptoms, and early referral to psycho-oncology services. These interventions may help strengthen psychological resilience, improve patients’ perception of treatment, and facilitate better physical and emotional adaptation during therapy. The systematic integration of quality-of-life assessments into clinical practice enables clinicians to tailor supportive and therapeutic strategies to the individual needs of each patient.

The changes observed in quality-of-life indicators during treatment reflect the combined effects of pelvic radiotherapy and concurrent capecitabine, rather than the impact of radiotherapy alone. This cumulative toxicity is especially relevant for gastrointestinal symptoms, fatigue, and general discomfort. Moreover, the absence of statistically significant differences between patients who received prior CAPOX chemotherapy and those who did not suggests that the chemoradiotherapy protocol was well tolerated in our cohort. This finding is consistent with the post hoc analysis by Kosmala et al. (2021) in the CAO/ARO/AIO-04 trial, which showed that the addition of oxaliplatin was not associated with decreased overall quality of life in disease-free patients, despite the risk of neurotoxicity [106].

This study presents several limitations that should be considered when interpreting the results. First, although the sample size was adequate for statistical analysis, the generalizability of the conclusions to broader populations may be limited. Second, quality-of-life assessment relied exclusively on self-reported responses, which may have introduced a degree of subjectivity in the evaluation of symptoms and functioning. The relatively small final sample and unequal subgroup distribution limited the feasibility of multivariate regression analysis. Future studies with larger cohorts are needed to better evaluate the contribution of independent variables such as age, comorbidities, disease stage, and treatment modality to quality-of-life outcomes. The absence of post-treatment follow-up limits the assessment of the long-term impact on quality of life and psychosocial adjustment. Future studies should include extended longitudinal evaluations and targeted interventions to support the emotional and social adaptation of patients, particularly those with a stoma or multiple comorbidities.

## 5. Conclusions

This study demonstrates a decline in physical, emotional, social, and role functioning, along with worsening fatigue, pain, and gastrointestinal and urinary symptoms during radiotherapy with concurrent capecitabine. Despite symptom progression, global quality of life remained relatively stable, suggesting psychological adaptation. Patients without a stoma reported better outcomes across most domains, indicating a negative impact of stoma presence on quality of life. No significant differences were observed based on prior chemotherapy, suggesting good tolerability of the combined regimen. Stoma creation was associated with older age, male sex, lower education, and advanced disease stage. These findings support the need for systematic quality-of-life monitoring and personalized treatment planning. Integrating patient-reported outcomes into clinical care can guide therapeutic decisions and enhance supportive interventions.

## Figures and Tables

**Table 1:** Distribution of patients according to demographic and clinicopathological characteristics.

Demographic Characteristics		Number of Patients	%
Age	18–49	6	9.4
	50–69	41	64.1
	≥70	17	26.5
Sex	Male	43	67.2
	Female	21	32.8
Residence area	Urban	36	56.3
	Rural	28	43.7
Education level	Primary	15	23.4
	Secondary	39	60.9
	Higher	10	15.7
Smoker	No	34	53.1
	yes	30	46.9
Alcohol consumption	Rare/occasional	23	35.9
	Moderate	34	53.2
	Chronic	7	10.9
**Clinicopathological Characteristics**		**Number of Patients**	**%**
Associated pathologies	Cardiovascular (CV)	22	34.4
	CV + Diabetes	7	10.9
	Diabetes	3	4.7
	Other neoplasms for over 2 years	3	4.7
	Obesity	3	4.7
	Liver diseases	2	3.1
	No other pathologies	24	37.5
Tumor location	Lower rectum	20	31.2
	Middle rectum	27	42.2
	Upper rectum	17	26.6
Tumor differentiation degree	G1	12	18.8
	G2	41	64.1
	G3	11	17.2
Staging	IIIA	24	37.5
	IIIB	26	40.6
	IIIC	3	4.7
	IV	9	14.1
	IIA	2	3.1
CAPOX chemotherapy	Yes	51	79.7
	No	13	20.3

**Table 2:** Analysis of functional scale scores at three assessment time points.

Functional Scale	Baseline Mean/CI 95%	After 15 RT Sessions Mean/CI 95%	After Completing RT Sessions Mean/CI 95%	*p*-Value	ANOVA Statistic	Eta Squared (η^2^)
Physical functioning	8.31/ [7.83, 8.8]	8.88/ [8.2, 9.55]	10.16/ [9.37, 10.95]	0.00054	7.83	0.0765
Social role fulfillment	3.86/ [3.4, 4.32]	4.02/ [3.58, 4.45]	4.7/ [4.27, 5.14]	0.021256	3.93	0.0399
Emotional functioning	7.78/ [7.14, 8.42]	8.17/ [7.47, 8.87]	9.55/ [8.81, 10.29]	0.001304	6.88	0.0679
Social functioning	3.3/ [2.95, 3.64]	3.67/ [3.28, 4.06]	4.3/ [3.88, 4.71]	0.001588	6.67	0.0659
Total functioning scale	23.25/ [21.91, 24.59]	24.73/ [23.02, 26.45]	28.7/ [26.67, 30.74]	0.000055	10.34	0.0986

**Table 3:** Analysis of symptom scale scores at three assessment time points.

Functional Scale	Baseline Mean/CI 95%	After 15 RT Sessions Mean/CI 95%	After Completing RT Sessions Mean/CI 95%	*p*-Value	ANOVA Statistic	Eta Squared (η^2^)
Fatigue	5.91/ [5.41, 6.41]	6.19/ [5.65, 6.72]	7.48/ [6.85, 8.12]	0.00023	8.75	0.0848
Pain	4.69/ [4.22, 5.16]	5.98/ [5.46, 6.51]	7.3/ [6.78, 7.82]	<0.0001	25.67	0.2136
Loss of appetite and taste alteration	5.22/ [4.74, 5.7]	5.12/ [4.72, 5.53]	5.83/ [5.32, 6.33]	0.07606	2.61	0.0269
Nausea and vomiting	2.98/ [2.64, 3.33]	2.92/ [2.62, 3.22]	3.34/ [3.0, 3.69]	0.16272	1.83	0.019
Urinary symptoms	6.89/ [6.36, 7.42]	7.89/ [7.36, 8.42]	9.42/ [8.24, 10.61]	0.00012	9.48	0.0912
Gastrointestinal symptoms	10.2/ [9.43, 10.97]	10.77/ [10.09, 11.44]	12.98/ [12.12, 13.85]	<0.0001	13.92	0.1284
Hair loss	1.17/ [1.04, 1.3]	1.22/ [1.08, 1.35]	1.25/ [1.08, 1.42]	0.7537	0.28	0.003
Total symptom scale	37.06/ [34.58, 39.55]	40.09/ [37.91, 42.28]	47.61/ [44.28, 50.94]	<0.0001	15.42	0.1403

**Table 4:** Analysis of clinico-demographic variables associated with stoma presence (χ^2^ and *p*-value).

Patient Variable	χ^2^ Value	Degrees of Freedom (df)	χ^2^ Threshold	*p*-Value
Chemotherapy (yes/no)	0.4	1	3.84	0.5261
Disease stage	14.19	4	9.49	0.0067
Degree of differentiation	4.13	2	5.99	0.127
Tumor localization	1.2	2	5.99	0.549
Associated pathologies	6.43	6	12.59	0.3768
Alcohol consumption	0.56	2	5.99	0.7547
Smoking	0	1	3.84	1
Education	9.7	2	5.99	0.0078
Residence area	0.32	1	3.84	0.5721
Sex	8.66	1	3.84	0.0032
Age	64.42	2	5.99	0.0004

**Table 5:** Mean scores of patients with and without a stoma for group-specific questions at three assessment time points.

Scale		Baseline Mean/CI 95%	After 15 RT Sessions Mean/CI 95%	After Completing RT Sessions Mean/CI 95%	*p*-Value	ANOVA Statistic	Eta Squared (η^2^)
Patients without stoma	GI symptoms	7.14/ [6.55, 7.72]	7.96/ [7.25, 8.68]	9.35/ [8.52, 10.19]	0.000151	9.34	0.1107
	Emotional functioning	1.94/ [1.68, 2.2]	2.12/ [1.87, 2.36]	2.39/ [2.12, 2.66]	0.05345	2.99	0.0383
	Pain	1.43/ [1.25, 1.62]	2.22/ [1.97, 2.46]	2.75/ [2.53, 2.96]	<0.0001	36.01	0.3244
	Global QoL	10.24/ [9.49, 10.98]	9.41/ [8.59, 10.23]	9.16/ [8.5, 9.81]	0.112498	2.22	0.0287
Patients with stoma	GI symptoms	6.23/ [5.34, 7.12]	7.92/ [6.43, 9.42]	10.38/ [8.99, 11.78]	0.000331	10.09	0.3593
	Emotional functioning	2.38/ [1.91, 2.86]	2.23/ [1.68, 2.78]	3.15/ [2.72, 3.59]	0.028437	3.94	0.1795
	Pain	1.62/ [1.26, 1.97]	2.23/ [1.68, 2.78]	2.31/ [1.79, 2.82]	0.1057	2.39	0.1174
	Stoma care difficulties	2.0/ [1.5, 2.5]	2.31/ [1.7, 2.91]	2.38/ [1.97, 2.8]	0.549564	0.61	0.0327
	Global QoL	10.31/ [8.6, 12.02]	10.31/ [8.81, 11.8]	9.46/ [7.96, 10.96]	0.69241	0.37	0.0202

**Table 6:** Analysis of mean functioning scores between patients with and without chemotherapy at three assessment time points.

Assessment Time Point	Functioning Scale	Mean Score (SD)		t-Statistic	*p*-Value
		Patients with Chemotherapy	Patients Without Chemotherapy		
Baseline	Physical functioning	8.14 (1.81)	9.00 (2.48)	−1.18	0.26
	Social role fulfillment	3.86 (1.92)	3.85 (1.77)	0.03	0.98
	Emotional functioning	7.90 (2.74)	7.31 (1.93)	0.9	0.38
	Social functioning	3.33 (1.48)	3.15 (1.07)	0.5	0.62
	Total functioning scale	23.24 (5.52)	23.31 (5.51)	−0.04	0.97
After 15 RT sessions	Physical functioning	8.98 (2.89)	8.46 (2.22)	0.7	0.49
	Social role fulfillment	4.04 (1.84)	3.92 (1.55)	0.23	0.82
	Emotional functioning	8.29 (3.05)	7.69 (1.97)	0.87	0.39
	Social functioning	3.69 (1.68)	3.62 (1.26)	0.17	0.87
	Total functioning scale	25.00 (7.24)	23.69 (6.05)	0.67	0.51
After completing RT sessions	Physical functioning	10.04 (3.25)	10.62 (3.20)	−0.58	0.57
	Social role fulfillment	4.69 (1.85)	4.77 (1.54)	−0.17	0.87
	Emotional functioning	9.47 (3.12)	9.85 (2.64)	−0.44	0.66
	Social functioning	4.25 (1.72)	4.46 (1.61)	−0.41	0.69
	Total functioning scale	28.45 (8.48)	29.69 (7.75)	−0.51	0.62

**Table 7:** Analysis of mean symptom scores between patients with and without chemotherapy at three assessment time points.

Assessment Time Point	Symptom Scale	Mean score (SD)		t-Statistic	*p*-Value
		Patients with Chemotherapy	Patients Without Chemotherapy		
Baseline	Fatigue	5.29 (2.19)	5.62 (1.84)	−0.58	0.57
	Pain	8.56 (1.39)	9.15 (1.13)	−0.62	0.54
	Loss of appetite and taste alteration	5.74 (1.88)	5.31 (1.66)	0.57	0.57
	Nausea and vomiting	2.76 (1.02)	2.92 (0.48)	−0.26	0.79
	Urinary symptoms	7.85 (1.47)	7.38 (1.14)	0.63	0.53
	Gastrointestinal symptoms	13.32 (2.91)	13.85 (2.73)	−0.53	0.6
	Hair loss	2.62 (1.00)	2.46 (0.69)	0.32	0.75
	Total symptom scale	46.15 (5.76)	46.69 (4.98)	−0.24	0.81
After 15 RT sessions	Fatigue	5.10 (2.08)	5.38 (1.76)	−0.39	0.7
	Pain	8.74 (1.41)	8.77 (1.32)	−0.04	0.97
	Loss of appetite and taste alteration	6.02 (1.60)	5.54 (1.39)	0.56	0.58
	Nausea and vomiting	2.98 (0.99)	3.15 (0.76)	−0.25	0.81
	Urinary symptoms	7.66 (1.58)	7.62 (1.66)	0.04	0.97
	Gastrointestinal symptoms	13.54 (2.92)	13.92 (2.67)	−0.3	0.76
	Hair loss	2.50 (0.94)	2.31 (0.76)	0.33	0.74
	Total symptom scale	46.54 (4.77)	46.69 (5.07)	−0.07	0.94
After completing RT sessions	Fatigue	5.42 (2.27)	5.54 (2.50)	−0.15	0.88
	Pain	8.88 (1.56)	8.69 (1.80)	0.16	0.87
	Loss of appetite and taste alteration	6.12 (2.01)	5.92 (2.26)	0.2	0.85
	Nausea and vomiting	3.08 (4.53)	2.92 (1.59)	0.22	0.83
	Urinary symptoms	7.88 (1.61)	7.46 (2.13)	0.46	0.65
	Gastrointestinal symptoms	13.77 (2.32)	13.54 (2.63)	0.17	0.87
	Hair loss	2.69 (1.00)	2.38 (0.96)	0.51	0.61
	Total symptom scale	47.85 (8.84)	46.92 (8.83)	0.29	0.77

## Data Availability

The original contributions presented in this study are included in the article material. Further inquiries can be directed to the corresponding authors.
